# Pan-Cancer Analysis of NOP2 Reveals Its Prognostic Relevance and Association With the Tumor Immune Microenvironment

**DOI:** 10.14740/wjon2739

**Published:** 2026-05-08

**Authors:** Chuan Kun Yang, Wei Li, Shuang Liu, Yue Qi Wang, Su Su Luo, Wei Xiang Wang

**Affiliations:** a Center of Clinical Laboratory Medicine, Zhongda Hospital, Southeast University, Nanjing 210009, China; b Department of Clinical Research Center, Children’s Hospital of Nanjing Medical University, Nanjing 210008, China; c Department of Laboratory Medicine, Zhongda Hospital, Medical School of Southeast University, Nanjing 210009, China; d The Third Department of Hepatic Surgery, Shanghai Eastern Hepatobiliary Surgery Hospital, Shanghai 200438, China; e Moores Cancer Center, School of Medicine, University of California San Diego, La Jolla, CA 92093, USA; f Department of Infectious Disease Control and Prevention, Nanjing Municipal Center of Disease Control and Prevention, Nanjing 210009, China

**Keywords:** NOP2, Pan-cancer, Prognosis, Immune infiltration, Bioinformatics

## Abstract

**Background:**

RNA 5-methylcytosine (m^5^C) modification has emerged as an important layer of epigenetic regulation in cancer biology. NOP2 (also known as NSUN1), a nucleolar RNA methyltransferase involved in ribosomal RNA methylation and ribosome biogenesis, has been reported to promote tumor progression in several individual malignancies. However, the expression pattern, prognostic relevance, and immune-related features across multiple cancer types remain incompletely understood.

**Methods:**

We conducted an integrative pan-cancer analysis of NOP2 across 33 tumor types using publicly available datasets and web tools, including The Cancer Genome Atlas (TCGA), the Human Protein Atlas (HPA), Gene Expression Profiling Interactive Analysis 2 (GEPIA2), cBioPortal for Cancer Genomics (cBioPortal), the University of Alabama at Birmingham Cancer data analysis portal (UALCAN), Search Tool for the Retrieval of Interacting Genes/Proteins (STRING), and SangerBox. NOP2 expression differences between tumor and normal tissues were evaluated at both mRNA and protein levels. Survival analyses were conducted using GEPIA2, and genetic alterations were characterized via cBioPortal. The relationships between NOP2 expression and the tumor immune microenvironment were assessed by estimating tumor-infiltrating immune cell proportions with Cell-type Identification By Estimating Relative Subsets Of RNA Transcripts (CIBERSORT), followed by correlation analyses with immune cell infiltration levels, immune checkpoint-related genes, tumor mutational burden (TMB), and microsatellite instability (MSI). Finally, protein-protein interaction and co-expression analyses were conducted to identify NOP2-interacting and NOP2-correlated genes, which were subjected to functional enrichment analyses to infer potential biological pathways associated with NOP2.

**Results:**

NOP2 mRNA was significantly upregulated in 20 of 33 tumor types compared with normal tissues (|log_2_FC| ≥ 1, q < 0.01), with protein-level overexpression confirmed in most cancers examined (P < 0.001). High NOP2 expression was associated with shorter overall survival (OS) in 11 cancer types and poorer disease-free survival (DFS) in 13 cancer types (P < 0.05), including renal cell carcinomas, hepatocellular carcinoma, lower-grade glioma, and lung adenocarcinoma. Genetic alterations of NOP2 were most frequent in ovarian cancer (6.1%) and patients with NOP2 genetic alterations were associated with poorer OS, DSS, and PFS in the cBioPortal cohort analysis. NOP2 expression correlated significantly with mast cell infiltration in 24 cancer types and CD8^+^ T-cell infiltration in 19 cancer types (P < 0.05). Positive correlations between NOP2 expression and TMB were observed in 14 of 15 cancer types showing significant associations. Functional enrichment analysis confirmed associations with ribosome biogenesis and RNA processing pathways.

**Conclusion:**

This pan-cancer analysis shows that NOP2 is broadly dysregulated and associated with adverse survival and immune-related features across multiple human cancers. However, given the exploratory and largely univariate nature of the present study, its independent prognostic value and clinical utility require further validation in cancer-specific cohorts with multivariable and comparative predictive analyses.

## Introduction

Cancer remains a leading cause of morbidity and mortality worldwide. Although advances in early detection, surgical techniques, and systemic therapies have improved outcomes for some patients, the prognosis of many patients with malignancies, particularly those with advanced-stage disease, remains poor [1, 2]. In recent years, immunotherapy, especially immune checkpoint blockade, has revolutionized cancer treatment. However, only a subset of patients derive durable benefit, highlighting substantial heterogeneity in therapeutic response and underscoring the need for biomarkers that can predict prognosis and reflect the tumor immune microenvironment [3, 4].

Accumulating evidence indicates that epigenetic and post-transcriptional regulatory mechanisms play critical roles in tumor progression and immune regulation [5]. Among these, RNA modifications have emerged as important modulators of gene expression beyond transcriptional control [6]. One of the most prevalent RNA modifications, 5-methylcytosine (m^5^C), is involved in RNA stability, processing, and translation [7]. Dysregulation of m^5^C-related enzymes has been implicated in aberrant cell proliferation, metabolic reprogramming, and malignant transformation [8, 9]. Nevertheless, the oncogenic and immunological roles of many m^5^C regulators across diverse cancer types remain incompletely characterized.

NOP2 (also known as NSUN1 or p120) is a nucleolar RNA methyltransferase that catalyzes m^5^C modification of ribosomal RNA and plays a key role in ribosome biogenesis and cell cycle progression [10]. Previous studies have reported that NOP2 is aberrantly overexpressed in several malignancies, including hepatocellular carcinoma, ovarian cancer, lung cancer, and colorectal cancer, where it promotes tumor cell proliferation, invasion, and metabolic reprogramming [11–14]. Mechanistically, NOP2-mediated m^5^C modification contributes to ribosome assembly and oncogenic signaling, suggesting that NOP2 may function as a driver of malignant phenotypes [10, 11, 14]. Importantly, early classical studies had identified NOP2 as a proliferation-associated nucleolar protein with tumorigenic potential: forced expression of human NOP2 enhanced NIH/3T3 cell growth and conferred tumorigenicity, while high NOP2 expression was associated with poor prognosis in stage I lung adenocarcinoma [15, 16]. In hematologic malignancies, NOP2 was shown to be expressed in HL-60 and MOLT-4 leukemic cells in a cell cycle-dependent manner, and its expression became undetectable after induction of myeloid differentiation in HL-60 cells, supporting a close association with the proliferative state of leukemic cells [17]. In addition, NOP2 was identified as a direct target of granulocyte colony-stimulating factor (G-CSF) signaling during myeloid differentiation [18].

More recently, NOP2/NSUN1 was shown to regulate ribosome biogenesis not only through its canonical methyltransferase-related activity, but also through non-catalytic complex formation with box C/D snoRNPs, indicating that its biological functions extend beyond rRNA m^5^C modification alone [10]. Accumulating evidence suggests that the oncogenic functions of NOP2 are not restricted to ribosome biogenesis. In several tumor types, NOP2 has been reported to promote malignant progression by regulating the m^5^C modification and stability of non-ribosomal transcripts, such as c-Myc, EZH2, LMNB2, and APOL1, thereby contributing to metabolic reprogramming, epithelial-mesenchymal transition, and oncogenic signaling pathways [11–14]. Together, these findings support NOP2 as a biologically important proliferation-related and tumor-promoting factor.

Based on these biological observations, recent pan-cancer and cancer-specific studies have increasingly examined its broader clinical and immunological relevance across human malignancies. Fang et al integrated bulk ribonucleic acid sequencing (RNA-seq) and single-cell RNA-seq analyses and demonstrated that NOP2 is broadly overexpressed across multiple cancers, with significant associations with immune-cell distribution, methylation and phosphorylation features, and immune checkpoint-related characteristics [19]. Liu et al conducted a comprehensive pan-cancer analysis of NOP2 across 33 tumor types and highlighted its associations with prognosis, tumor microenvironment features, immune modulators, and immunotherapeutic response [20]. In a cancer-specific context, Wang et al further demonstrated that NOP2 expression in clear cell renal cell carcinoma was associated with survival outcomes, microsatellite instability (MSI), tumor mutational burden (TMB), and immune characteristics [21]. These studies have provided important evidence supporting the involvement of NOP2 in tumor progression and cancer-related immune biology.

However, despite these advances, several important aspects of NOP2 biology remain insufficiently explored. In particular, integrating complementary transcriptomic and proteomic datasets, including The Cancer Genome Atlas (TCGA) and GTEx-based expression resources, may provide a more robust assessment of its tumor-versus-normal expression landscape across cancer types. In addition, the relationship between NOP2 and broader epitranscriptomic regulatory systems, especially RNA modification-related genes involved in the m^1^A, m^5^C, and m^6^A pathways, has not been systematically examined, and its associations with immune checkpoint genes (ICPGs) also warrant further clarification. Therefore, rather than claiming a wholly novel discovery of NOP2 dysregulation in cancer, our study was designed as an integrative and complementary pan-cancer analysis to validate, refine, and extend previous observations. Using multiple public datasets and analytical platforms, we systematically evaluated the expression landscape, prognostic relevance, and tumor immune microenvironment associations of NOP2 across 33 human malignancies, while further exploring its potential links with ICPGs and other RNA modification-related regulators.

## Materials and Methods

### Data sources and preprocessing

Transcriptomic data for pan-cancer analysis were obtained from the TCGA database, comprising RNA sequencing data for 33 tumor types, including tumor samples and available adjacent normal tissues, with a total of 11,315 samples. Because TCGA primarily contains tumor samples and some cancer types lack sufficient adjacent normal tissues, RNA-seq data from normal tissues in the GTEx dataset were incorporated to improve the robustness of tumor-versus-normal expression analyses.

The RNA-seq data from TCGA tumor samples were extracted and normalized to transcripts per million (TPM) values. To enable comparative expression analysis of NOP2 between tumor and normal tissues, TPM values were log_2_-transformed as log_2_ (TPM + 0.001). Differential expression analysis of NOP2 was performed with false discovery rate (FDR) correction for multiple testing, and |log_2_ fold change| ≥ 1 with an adjusted q-value < 0.01 was considered statistically significant. Protein expression and phosphorylation levels of NOP2 were further analyzed using UALCAN data analysis portal [22]. In addition, NOP2 expression patterns across different pathological tumor stages were evaluated.

### Protein expression analysis

Protein expression of NOP2 was evaluated using the HPA database [23], which provides comprehensive information on protein distribution in human tissues and cells. Representative immunohistochemistry (IHC) images were retrieved from the HPA database to examine NOP2 protein expression patterns in tumor tissues and corresponding normal tissues.

### Survival analysis

The prognostic value of NOP2 expression was assessed using the GEPIA2 platform through the “Survival Analysis” module [24]. Overall survival (OS) and disease-free survival (DFS) were compared between high- and low- NOP2 expression groups across different cancer types using the Kaplan–Meier method. Statistical significance was evaluated using the log-rank test. In addition, forest plot was generated to summarize the association between NOP2 expression and prognosis across 33 cancer types. Univariate survival analysis was performed to calculate the hazard ratio (HR) and its corresponding 95% confidence interval.

### Genetic alteration analysis

Genetic alterations in NOP2 were analyzed using cBioPortal for Cancer Genomics [25]. Mutation and copy number alteration data from TCGA pan-cancer cohorts were used to evaluate alteration frequency, copy number alterations (CNAs), and mutation types of NOP2 across different tumor types. Survival differences, including OS, progression-free survival (PFS), and DSS, were further analyzed between patients with and without NOP2 genetic alterations across the TCGA pan-cancer cohort. Kaplan–Meier survival curves were generated, and statistical significance was assessed using the log-rank test based on TCGA data.

### Immune microenvironment analysis

The CIBERSORT algorithm (v1.0.3) was used to estimate the relative proportions of 22 tumor-infiltrating immune cells (TIICs) in tumor tissues via the SangerBox platform [26, 27]. The LM22 leukocyte gene signature matrix (2015 version) was used as the reference dataset, batch effect correction was applied, and the number of permutations was set to 1,000. Samples with CIBERSORT P values < 0.05 were considered to have reliable immune infiltration estimates and were included in subsequent analyses. The evaluated immune cells populations included dendritic cells (DCs), activated DCs (aDCs), B cells, CD8^+^ T cells, immature DCs (iDCs), macrophages, mast cells, neutrophils, natural killer (NK) cells, and other immune subsets.

Spearman correlation analyses were performed to evaluate cross-sectional associations between NOP2 expression and various ICPGs, TMB, MSI, neoantigen load (NEO), and other tumor microenvironment biomarkers. Correlation results were visualized using bubble plots, in which bubble size represented the magnitude of the correlation coefficient, and color indicated statistical significance. Additionally, the Tumor and Immune System Interaction DataBase (TISIDB) [28] was used to analyze the association between NOP2 expression and immune and molecular subtypes across human cancers. Differences in NOP2 expression among subtype groups were assessed using the Kruskal–Wallis test. Data were accessed on September 29, 2023.

### Functional enrichment and protein-protein interaction (PPI) network analysis

PPI network analysis was performed using the STRING database [29]. The minimum required interaction score was set to low confidence, network edges were defined based on evidence, interaction sources were limited to experiments, and the maximum number of interactors was restricted to 50. Under these criteria, the top 50 proteins interacting with NOP2 were retrieved.

Genes with expression patterns similar to NOP2 were identified using the “Similar Gene Detection” module in GEPIA2, yielding 100 NOP2-correlated genes. A Venn diagram was generated to identify overlapping genes between NOP2-interacting proteins and NOP2-correlated genes, resulting in 17 shared genes. Gene Ontology (GO) and Kyoto Encyclopedia of Genes and Genomes (KEGG) pathway enrichment analyses were subsequently performed on the combined set of 133 genes. Gene ID conversion was conducted using the *org.hs.eg.db* package, and enrichment analyses were carried out using the *clusterProfiler* package. The whole annotated human genome was used as the background gene set for enrichment analysis. Data visualization was performed with *ggplot2*. All analyses were conducted using R software (version R-4.1.0, 64-bit, Vienna, Austria).

### Ethical approval

This article does not involve any studies with human participants or animals conducted by any of the authors.

## Results

### Differential expression of NOP2 in pan-cancer

**Figure 1:**
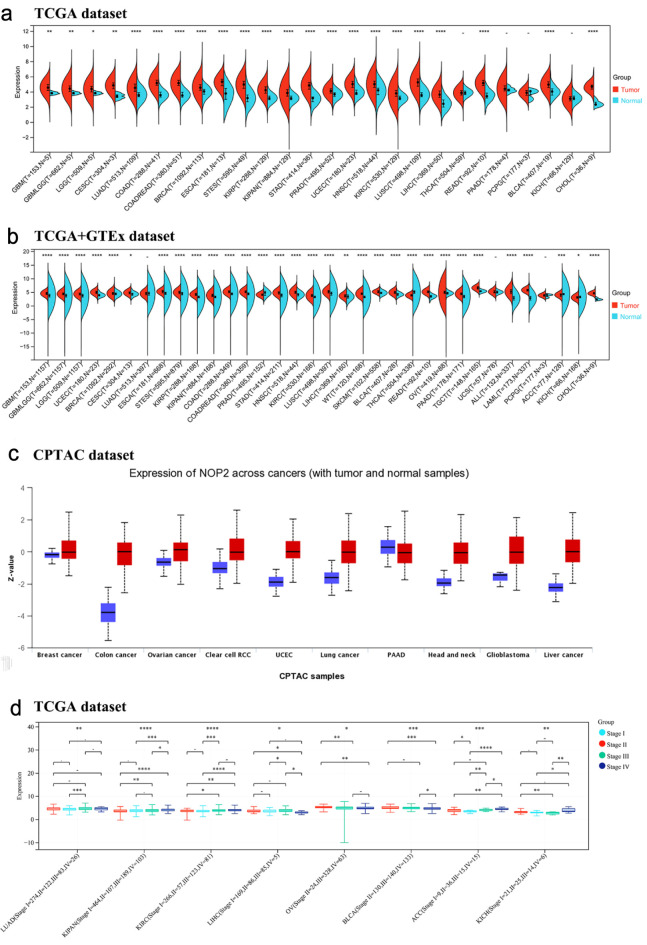
Expression of NOP2 mRNA in pan-cancer tissues. (a) Expression of NOP2 mRNA in 33 tumors in the TCGA database. (b) Expression of NOP2 mRNA in 33 tumors from TCGA-GTEx samples. (c) Total protein expression levels of NOP2 in tumor tissues, including breast cancer, colon cancer, ovarian cancer, PAAD, as well as normal tissues. (d) Differential expression levels of NOP2 at different pathological stages in tumor tissues such as LUAD, KIRC, LIHC. ACC: adrenocortical carcinoma; BLCA: bladder urothelial carcinoma; BRCA: breast invasive carcinoma; CESC: cervical and endocervical cancers; CHOL: cholangiocarcinoma; COAD: colon adenocarcinoma; DLBC: lymphoid neoplasm diffuse large B-cell lymphoma; ESCA: esophageal carcinoma; GBM: glioblastoma multiforme; HNSC: head and neck squamous cell carcinoma; KICH: kidney chromophobe; KIRC: kidney renal clear cell carcinoma; KIRP: kidney renal papillary cell carcinoma; LAML: acute myeloid leukemia; LGG: brain lower grade glioma; LIHC: liver hepatocellular carcinoma; LUAD: lung adenocarcinoma; LUSC: lung squamous cell carcinoma; MESO: mesothelioma; OV: ovarian serous cystadenocarcinoma; PAAD: pancreatic adenocarcinoma; PCPG: pheochromocytoma and paraganglioma; PRAD: prostate adenocarcinoma; READ: rectum adenocarcinoma; SARC: sarcoma; SKCM: skin cutaneous melanoma; STAD: stomach adenocarcinoma; STES: stomach and esophageal carcinoma; TGCT: testicular germ cell tumors; THCA: thyroid carcinoma; THYM: thymoma; UCEC: uterine corpus endometrial carcinoma; UCS: uterine carcinosarcoma; UVM: uveal melanoma (Ns, P > 0.05; *P < 0.05; **P < 0.01; ***P < 0.001).

Analysis of TCGA tumor and adjacent normal tissue data showed that NOP2 expression was significantly elevated in multiple tumor types, whereas no significant difference was observed in only a small subset of cancers, including thyroid carcinoma (THCA), pancreatic adenocarcinoma (PAAD), pheochromocytoma and paraganglioma (PCPG), and kidney chromophobe (KICH) (Fig. 1a). After integration with GTEx normal tissue data, the overall pattern of NOP2 upregulation remained largely consistent, although several cancer types showed discrepant results between TCGA-only and TCGA-GTEx analyses (Fig. 1b).

NOP2 protein expression levels were further examined using Clinical Proteomic Tumor Analysis Consortium (CPTAC) datasets. Among the CPTAC cancer types examined, total NOP2 protein expression was significantly higher in tumor tissues than in normal tissues, except for PAAD (Fig. 1c).

The association between NOP2 expression and pathological stages was evaluated across cancer types. Significant stage-associated differences in NOP2 expression were observed in LUAD, pan-kidney cohort (KIPAN), kidney renal clear cell carcinoma (KIRC), LIHC, ovarian serous cystadenocarcinoma (OV), bladder urothelial carcinoma (BLCA), adrenocortical carcinoma (ACC), and KICH (Fig. 1d), while no significant differences were detected in other tumor types (Supplementary Material 1, wjon.elmerpub.com).

**Figure 2:**
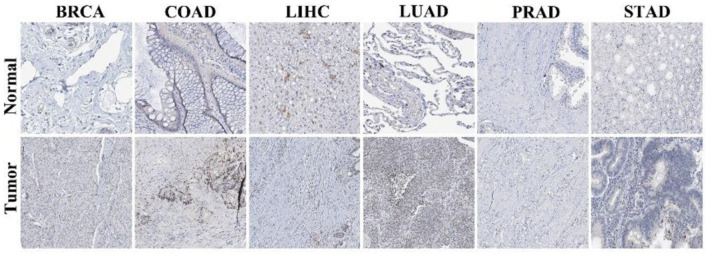
Immunohistochemical staining of NOP2 in normal and tumor tissues. Representative immunohistochemistry (IHC) images of NOP2 expression in normal and corresponding tumor tissues obtained from the Human Protein Atlas (HPA) database.

In addition, IHC data from the Human Protein Atlas (HPA) further demonstrated higher NOP2 protein expression in tumor tissues compared with corresponding normal tissues in several cancer types, including breast invasive carcinoma (BRCA), colon adenocarcinoma (COAD), liver hepatocellular carcinoma (LIHC), LUAD, prostate adenocarcinoma (PRAD), and stomach adenocarcinoma (STAD) (Fig. 2).

### Prognostic significance of NOP2 across cancers

**Figure 3:**
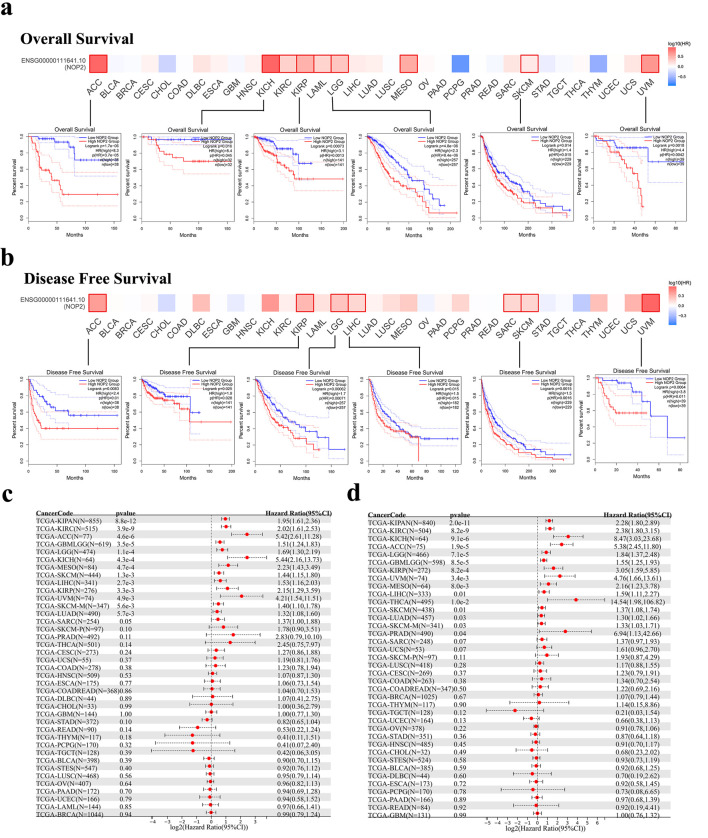
Association between NOP2 expression and survival outcomes in TCGA cancers. (a) Kaplan–Meier analysis of overall survival (OS) according to NOP2 expression levels in ACC, KICH, KIRP, LGG, SKCM, and UVM. (b) Kaplan–Meier analysis of disease-free survival (DFS) according to NOP2 expression levels in ACC, KIRP, LGG, LIHC, SKCM, and UVM. (c) Forest plot summarizing the association between NOP2 expression and OS across 33 cancer types. (d) Forest plot summarizing the association between NOP2 expression and DFS across 33 cancer types. ACC: adrenocortical carcinoma; KICH: kidney chromophobe; KIRP: kidney renal papillary cell carcinoma; LGG: brain lower grade glioma; LIHC: liver hepatocellular carcinoma; SKCM: skin cutaneous melanoma; UVM: uveal melanoma.

The prognostic value of NOP2 expression was evaluated across 33 cancer types using GEPIA2. High NOP2 expression was significantly associated with shorter OS in multiple cancers, including ACC, KICH, KIRC, kidney renal papillary cell carcinoma (KIRP), acute myeloid leukemia (LAML), brain lower grade glioma (LGG), mesothelioma (MESO), skin cutaneous melanoma (SKCM), and uveal melanoma (UVM). In contrast, high NOP2 expression was significantly associated with prolonged OS in pheochromocytoma and paraganglioma (PCPG) (Fig. 3a).

Similarly, high NOP2 expression was associated with poorer DFS in multiple tumor types, again with prominent associations in ACC, KIRP, LGG, LIHC, sarcoma (SARC), SKCM, and UVM (Fig. 3b). Kaplan–Meier survival curves and forest plots were further employed to summarize the associations between NOP2 expression and OS and DFS across cancer types (Fig. 3c, d).

### Genetic alterations and phosphorylation profiles of NOP2 across cancers

**Figure 4:**
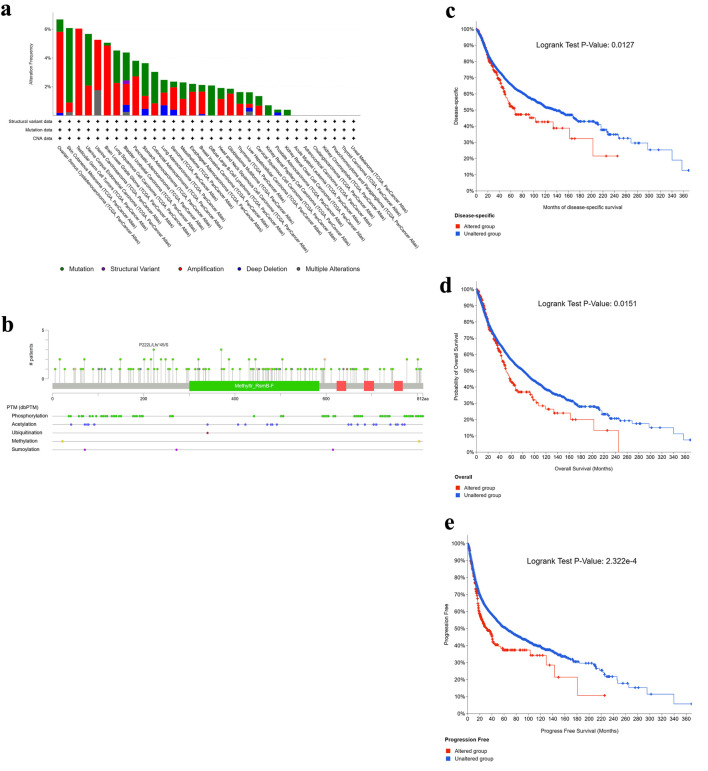
Genetic alteration landscape of NOP2 across cancers. (a) Alteration frequency of NOP2 across different tumor types. (b) Distribution of NOP2 mutation types, mutation sites, and affected cases. (c–e) Kaplan–Meier analyses illustrating the impact of NOP2 genetic alterations on disease-specific survival (DSS), overall survival (OS), and progression-free survival (PFS), respectively.

Genetic alteration analysis using cBioPortal showed that NOP2 was altered across multiple cancer types, with the highest alteration frequency observed in ovarian cancer (OV, 6.1%) (Fig. 4a). Mutations were the predominant alteration type in SKCM, uterine corpus endometrial carcinoma (UCEC), lung squamous cell carcinoma (LUSC), and STAD, whereas copy number amplification was more frequent in OV, testicular germ cell tumors (TGCT), uterine carcinosarcoma (UCS), and LGG. Detailed mutation types and sites are shown in Figure 4b. In the pooled survival analysis, patients harboring any NOP2 alteration had poorer OS, DSS, and PFS than unaltered cases (Fig. 4c–e). However, because amplification and mutation events were analyzed together without variant-level functional annotation, the observed survival association remains preliminary and should not be interpreted as evidence that all NOP2 alteration classes have equivalent biological or prognostic effects.

**Figure 5:**
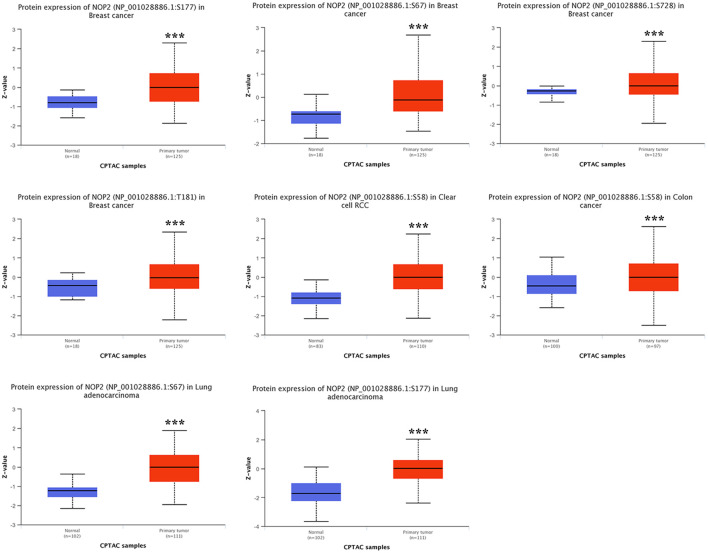
Comparison of NOP2 phosphorylation levels between tumor and normal tissues. NOP2 phosphorylation levels at specific sites in tumor and corresponding normal tissues based on CPTAC datasets across selected cancer types.

NOP2 phosphorylation levels were further evaluated using CPTAC dataset in breast cancer, clear cell renal cell carcinoma (ccRCC), colon cancer, and LUAD. Phosphorylation profiles of NOP2 in tumor and normal tissues are shown in Figure 5. In breast cancer, significant differences in phosphorylation levels at sites S67, S177, S728, and T181 were observed between tumor and normal tissues (P < 0.001). In LUAD, phosphorylation levels of S177 and S67 were higher in normal tissues compared to tumor tissues (P < 0.001). Similarly, phosphorylation at site S58 was significantly higher in normal tissues than in tumor tissues in both colon cancer and ccRCC (P < 0.001).

### Correlation between NOP2 and RNA modification gene expression across cancer

**Figure 6:**
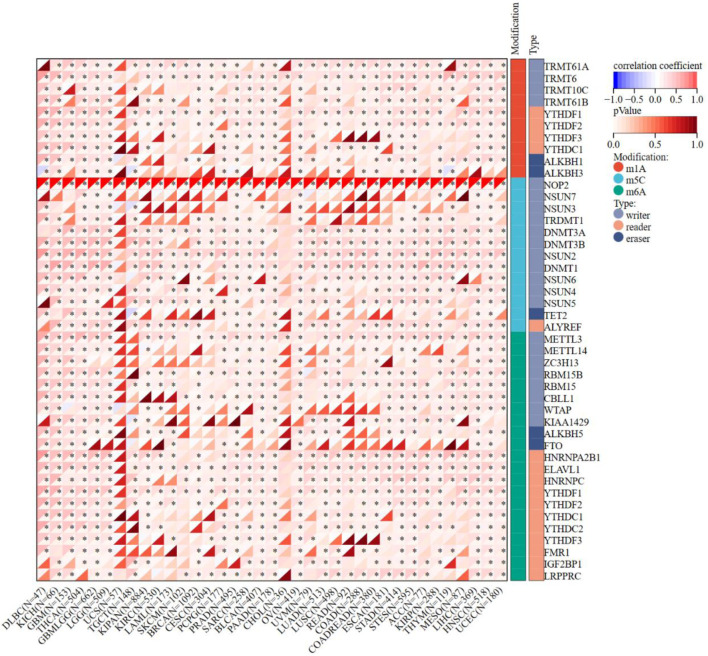
Correlation matrix of NOP2 expression and RNA modification-related gene expression. Correlation analysis between NOP2 expression and genes associated with RNA modifications, including m^1^A, m^5^C, and m^6^A pathways across cancers. *P < 0.05.

The expression of NOP2 and 44 genes related to m^1^A, m^5^C, and m^6^A RNA modification pathways was analyzed across TCGA pan-cancer datasets (Fig. 6). Overall, NOP2 showed predominantly positive correlations with RNA modification-related genes in most tumor types, including KICH, LGG, LUAD, and ACC, whereas negative correlations were relatively uncommon. This pattern suggests that NOP2 upregulation is broadly accompanied by coordinated activation of multiple epitranscriptomic regulatory programs across cancers. Detailed cancer-specific correlation patterns are shown in Figure 6.

### Immune- and molecular-subtype expression patterns of NOP2 across cancers

**Figure 7:**
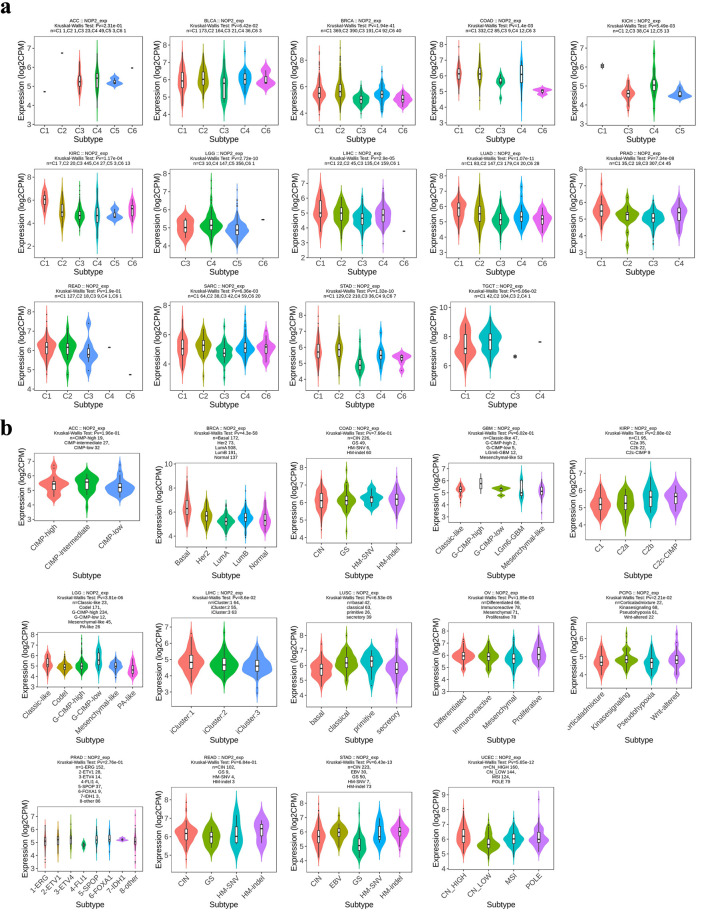
Distribution of NOP2 expression across immune and molecular subtypes of cancers. (a) Association between NOP2 expression and immune subtypes (C1–C6) across cancers. (b) Association between NOP2 expression and molecular subtypes across cancers.

Differences in NOP2 expression across immune and molecular subtypes were evaluated in multiple tumor types (Fig. 7). Significant variation in NOP2 expression across immune subtypes (C1–C6) were observed in several cancers, including BLCA, BRCA, COAD, KICH, KIRC, LGG, LIHC, LUAD, PRAD, SARC, STAD, and TGCT, whereas no significant differences were detected in a few tumor types such as ACC and rectum adenocarcinoma (READ). Similarly, NOP2 expression varied significantly across molecular subtypes in several cancers, including BRCA, KIRP, LGG, LIHC, LUSC, OV, PRAD, STAD, and UCEC. These findings suggest that NOP2 expression is associated with tumor immune and molecular heterogeneity in a cancer-type-dependent manner.

### Correlation between NOP2 expression and tumor immune microenvironment

**Figure 8:**
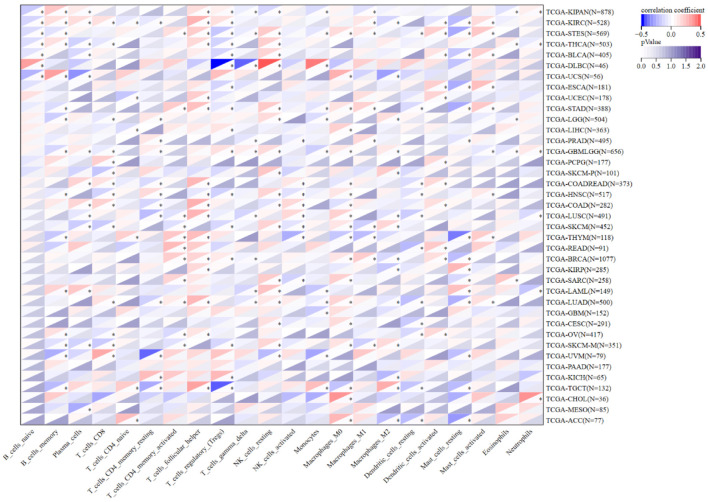
Correlation between NOP2 expression and infiltration of 22 immune cell types. Correlation analysis between NOP2 expression and the estimated infiltration levels of 22 tumor-infiltrating immune cell populations across cancers. *P < 0.05.

The association between NOP2 expression and immune cell infiltration was evaluated using the CIBERSORT algorithm across multiple cancer types. Cross-sectional analysis based on bulk RNA-seq data and CIBERSORT-estimated immune infiltration suggested that NOP2 expression was significantly associated with mast cell infiltration in 24 cancer types and CD8^+^ T-cell infiltration in 19 cancer types (Fig. 8). These findings suggest that NOP2 expression is significantly associated with immune infiltration patterns across cancers, particularly with immune cell subsets relevant to tumor immune activation and immune contexture. Nevertheless, the present analyses are cross-sectional and correlation-based, they do not support a causal inference that NOP2 directly regulates mast-cell infiltration or shapes the tumor immune microenvironment.

**Figure 9:**
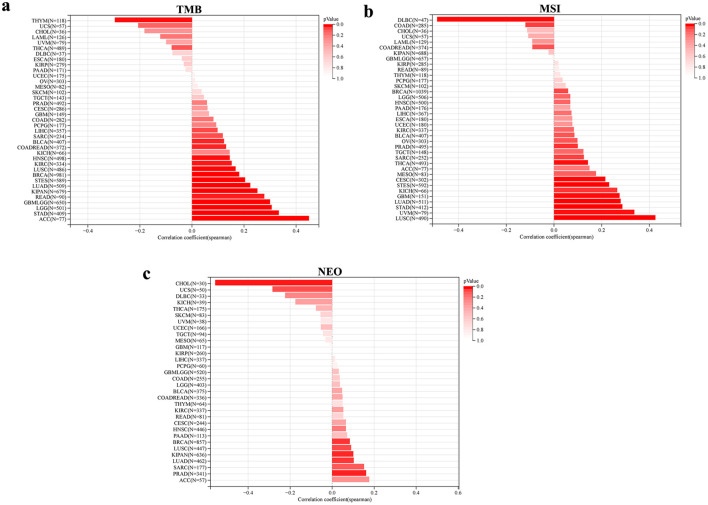
Association between genomic heterogeneity and NOP2 expression. (a) Correlation between NOP2 expression and tumor mutational burden (TMB) across cancer types. (b) Correlation between NOP2 expression and microsatellite instability (MSI) across cancer types. (c) Correlation between NOP2 expression and neoantigen load (NEO) across cancer types.

The associations between NOP2 expression and immunogenomic features, including TMB, MSI, and NEO, were further analyzed across cancers (Fig. 9). NOP2 expression was significantly associated with TMB in 15 cancer types, with positive correlations in most significant cases. In contrast, associations with MSI and NEO were less common and appeared to be more cancer-type dependent. These findings suggest that NOP2 expression is associated with multiple immunogenomic features across cancers, although these associations remain exploratory and hypothesis-generating.

**Figure 10:**
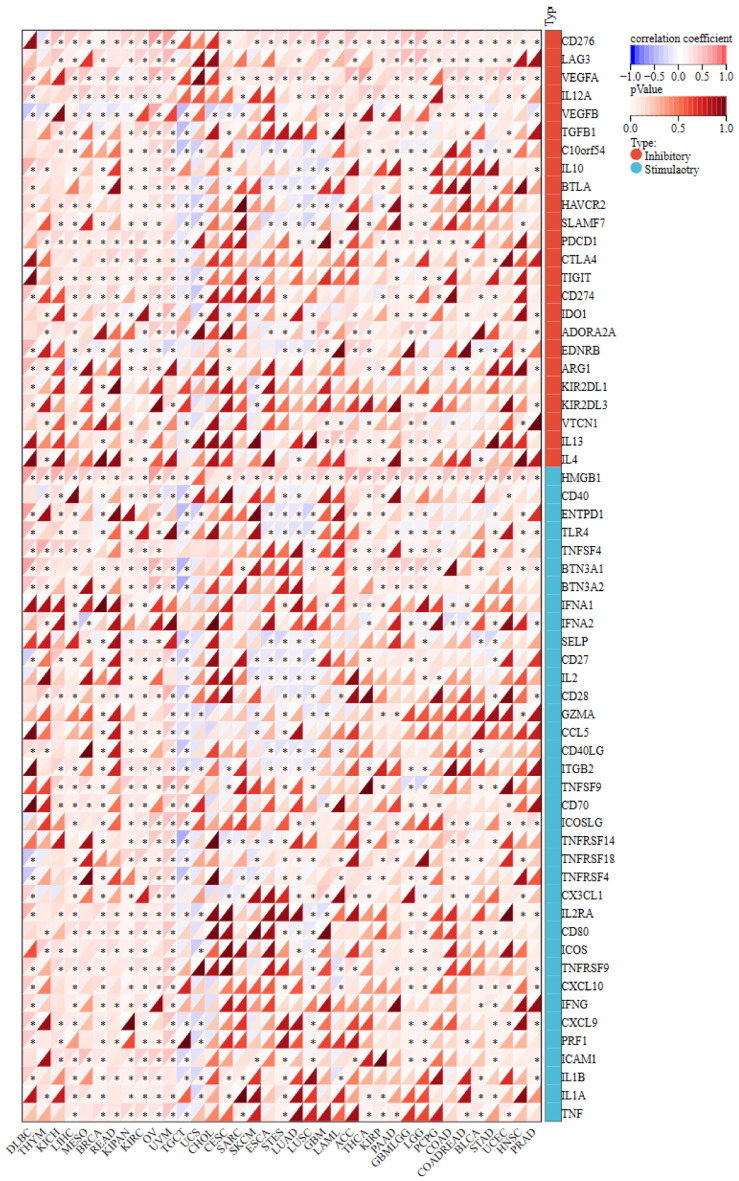
The correlation matrix of NOP2 expression and immune checkpoint-related gene expressions. *P < 0.05.

Correlations between NOP2 expression and 60 ICPGs were also assessed (Fig. 10). In most cancer types, including READ, PRAD, LIHC, LGG, PAAD, and BLCA, NOP2 expression was positively correlated with the expression of ICPGs, whereas negative correlations were observed in a smaller subset of cancers such as TGCT, UCS, and LUSC. These findings suggest that NOP2 expression may be associated with immune-related transcriptional features in multiple tumor types.

### Construction of the NOP2 interaction network and enrichment analysis

**Figure 11:**
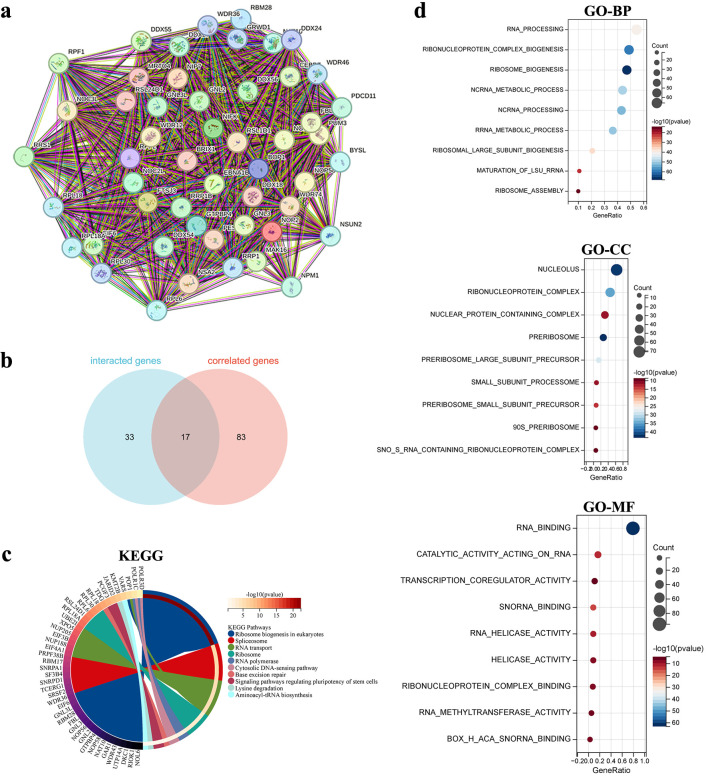
Functional enrichment analysis of NOP2-related genes. (a) Protein-protein interaction (PPI) network of 50 NOP2-interacting proteins derived from the STRING database. (b) Venn diagram showing the overlap between NOP2-interacting proteins and NOP2-correlated genes identified from GEPIA2. (c) KEGG pathways enrichment analysis based on NOP2-related genes. (d) GO enrichment analysis based on NOP2-related genes, including BP, CC, and MF.

To explore the functional characteristics of NOP2, 50 NOP2-interacting proteins were identified using the STRING database, and a PPI network was constructed (Fig. 11a). In parallel, 100 genes with the highest expression similarity to NOP2 were obtained from the GEPIA2 database (Supplementary Material 2, wjon.elmerpub.com). Seventeen overlapping genes were identified between the interacting proteins and NOP2-correlated genes (Fig. 11b).

GO and KEGG pathway enrichment analyses were subsequently performed using the combined set of 133 genes (Fig. 11c, d). GO enrichment analysis showed that these genes were mainly associated with RNA processing and ribosome biogenesis in the biological process category, nucleolus and ribonucleoprotein complex in the cellular component category, and RNA binding and transcription coregulator activity in the molecular function category. KEGG pathway analysis further indicated enrichment of pathways including ribosome biogenesis in eukaryotes, lysine degradation, and RNA transport (Fig. 11d).

## Discussion

In this study, we performed a comprehensive pan-cancer analysis to systematically evaluate the expression patterns, prognostic relevance, and immune-related associations of NOP2 across 33 human malignancies. Our findings demonstrate that NOP2 is broadly upregulated in multiple tumor types at both transcriptomic and proteomic levels, correlates with adverse survival outcomes in multiple cancer types, and exhibits meaningful associations with tumor immune microenvironment characteristics. By integrating multi-omics and immunological data from several public databases, our study further validates and extends previous observations regarding the biological and clinical significance of NOP2 in cancer.

The consistent upregulation of NOP2 across diverse malignancies aligns with its established function as a nucleolar RNA methyltransferase involved in ribosome biogenesis [10, 30]. Rapidly proliferating tumor cells typically exhibit increased ribosomal activity to support elevated protein synthesis demands, which may partly explain the preferential overexpression of NOP2 in malignant tissues [31]. The stage-associated expression patterns observed in LUAD, KIRC, LIHC, and several other cancers further suggest that NOP2 upregulation may accompany disease progression, although the cross-sectional nature of the present analyses does not allow causal inference.

Previous reports have shown that NOP2/NSUN1 is dysregulated in multiple malignancies and is associated with adverse prognosis and immune-related features. Fang et al integrated bulk RNA-seq and single-cell RNA-seq analyses and demonstrated that NOP2 is broadly overexpressed across multiple cancers, with significant associations with immune-cell distribution, methylation and phosphorylation features, and immune checkpoint-related characteristics [19]. Liu et al further highlighted the clinical and immunotherapeutic relevance of NOP2 in pan-cancer, including its associations with prognosis, tumor microenvironment features, immune modulators, and treatment response-related indicators [20]. In a cancer-specific setting, Wang et al showed that high NOP2 expression in ccRCC was significantly associated with poor OS and immune-related characteristics [21]. Therefore, our study does not provide the first evidence that NOP2 is aberrantly expressed or clinically relevant in cancer. Rather, the main value of the current study lies in its integrative extension of these earlier observations. Our expression analyses are largely consistent with previous pan-cancer studies, but the integration of TCGA and GTEx datasets allowed a more robust evaluation of tumor-versus-normal expression patterns and highlighted dataset-dependent differences for certain tumor types. In addition, we incorporated protein-level and phosphorylation-level analyses to complement transcriptomic findings. A notable contribution of the present study is the systematic analysis of correlations between NOP2 and RNA modification-related genes across cancers. The positive correlations between NOP2 and genes involved in m^1^A, m^5^C, and m^6^A modification pathways observed in most cancers suggest coordinated regulation of epitranscriptomic machinery in malignancy. This finding extends beyond previous analyses, as prior studies primarily focused on NOP2 in isolation without examining its relationship with the broader RNA modification landscape [19, 20]. The m^5^C modification pathway, of which NOP2 is a central component, intersects with m^6^A and m^1^A regulatory networks through shared targets and functional crosstalk [32, 33]. Whether NOP2 upregulation reflects a broader epitranscriptomic reprogramming in cancer or represents a selective advantage conferred by enhanced ribosomal RNA methylation remains an open question. The novelty of the present work should be regarded as incremental but meaningful, particularly in terms of cross-platform validation, biological contextualization, and extension of previous NOP2-centered analyses.

The prognostic significance of NOP2 across multiple cancers requires cautious interpretation. Although high NOP2 expression was consistently associated with shorter OS and DFS in renal cell carcinomas (KIRC, KIRP, KICH), ACC, lower-grade glioma, and hepatocellular carcinoma, these associations do not establish NOP2 as a causal driver of poor outcomes. Elevated NOP2 expression may instead reflect aggressive tumor biology, including enhanced proliferative activity and ribosome biogenesis, rather than serving as an independent prognostic determinant in all tumor contexts [12, 14, 34]. In ccRCC, our survival analysis findings are concordant with those reported by Wang et al, whereas our pan-cancer framework further indicates that the adverse prognostic association of NOP2 is not restricted to ccRCC but may extend to multiple renal and non-renal malignancies. Although an inverse association was observed in PCPG, most significant survival associations supported a link between elevated NOP2 expression and adverse outcomes. However, whether NOP2 has independent prognostic value or can improve risk stratification beyond established markers remains to be determined in future cancer-specific studies.

The genetic alteration landscape of NOP2 revealed notable heterogeneity across cancer types. Ovarian cancer exhibited the highest alteration frequency, predominantly driven by copy number amplification, consistent with the frequent chromosomal instability observed in high-grade serous carcinomas [35–37]. Our cBioPortal-based analysis showed that patients with any NOP2 alteration had poorer survival outcomes than unaltered cases, which should be interpreted cautiously because amplification and mutation events were analyzed together without variant-level functional annotation. We could not distinguish potentially activating alterations from rare inactivating or loss-of-function variants, which may have distinct prognostic implications. Therefore, the survival association observed in our study likely reflects the combined effects of heterogeneous alteration classes and tumor-type-specific genomic context, rather than a uniform adverse effect of all NOP2 alterations. These findings also suggest that NOP2 genomic dysregulation may provide prognostic information beyond expression changes alone. In addition, site-specific phosphorylation differences between tumor and normal tissues in several cancers, including breast cancer, LUAD, colon cancer, and ccRCC raise the possibility that post-translational regulation of NOP2 may also contribute to its oncogenic role [38, 39]. NOP2/NSUN1 has been implicated not only in 28S rRNA m^5^C methylation, but also in pre-rRNA processing and box C/D snoRNP assembly through non-catalytic functions [10]. Moreover, phosphorylation is a major post-translational mechanism that can influence protein localization, complex assembly, stability, and related functional states [40]. Although the functional significance of individual NOP2 phosphorylation sites has not yet been directly established, the differential phosphorylation identified at sites such as S67, S177, T181, S728, and S58 suggests that these residues may participate in regulating NOP2 function in a site-specific and tumor-context-dependent manner. Altered phosphorylation at these sites may plausibly affect NOP2 nucleolar localization, protein stability, RNA-binding capacity, or interactions with ribosome biogenesis-related partners, thereby modulating its methyltransferase-related and tumor-promoting activities. Therefore, the phosphoproteomic features of NOP2 identified in this study provide candidate sites for future investigation of its post-translational regulatory mechanisms in cancer. Further studies using site-directed mutagenesis, together with assays for subcellular localization, enzymatic activity, and tumor-related phenotypes, will be necessary to determine the precise biological significance of these phosphorylation events.

The immune-related findings of this study should be interpreted cautiously and are primarily descriptive and hypothesis-generating. The correlations between NOP2 expression and immune cell infiltration patterns were complex and context-dependent. Fang et al utilized single-cell RNA-seq data to demonstrate that NSUN1 is highly expressed in immune cells including T cells, B cells, and DCs within the tumor microenvironment [19]. Our bulk-tumor analysis complements these findings by showing that NOP2 expression was significantly associated with mast cell infiltration in 24 cancer types and with CD8^+^ T-cell infiltration in 19 cancer types. In several cancers, NOP2 expression showed inverse associations with cytotoxic immune populations such as CD8^+^ T cells, suggesting that NOP2-high tumors may harbor immunologically “cold” microenvironments [41, 42]. However, because these analyses were based on bulk RNA-seq data and CIBERSORT-estimated immune infiltration, they should be interpreted as cross-sectional associations rather than evidence that NOP2 directly regulates immune-cell recruitment, activation, or function. Further mechanistic studies, including *in vitro* and *in vivo* experiments, will be needed to clarify whether NOP2 plays a functional role in tumor immune modulation.

The associations between NOP2 expression and immunogenomic features were most evident for TMB, for which significant correlations were predominantly positive, which appears paradoxical given the generally adverse prognostic implications of high NOP2 expression. This pattern may reflect complex and context-dependent relationships between proliferative tumor biology and antitumor immunity. Specifically, elevated NOP2 expression may mark biologically aggressive tumors in which proliferative and tumor-promoting features outweigh any potential immune benefit associated with increased mutational burden. One possible explanation is that NOP2 upregulation accompanies genomic instability and hypermutation without necessarily enhancing effective tumor immunogenicity, as other immunosuppressive mechanisms may predominate in these tumors [43, 44].

The positive correlations between NOP2 expression and ICPG expression observed in multiple tumor types suggest that NOP2 may be linked to broader immune-related transcriptional features, and may warrant further investigation in the context of immune checkpoint blockade. Liu et al also reported positive correlations between NOP2 expression and TMB in multiple cancers, consistent with our findings [20]. However, because no consistent differences in NOP2 expression between immunotherapy responders and non-responders have been demonstrated across cohorts, the relationship between NOP2 and immunotherapy response should be interpreted cautiously and cannot currently be regarded as predictive.

Functional enrichment analysis of NOP2-associated genes confirmed expected roles in ribosome biogenesis and RNA processing while also revealing associations with RNA transport pathways. These findings are consistent with NOP2’s canonical function but do not exclude additional, less characterized activities. Recent evidence suggests that m^5^C modifications may regulate mRNA stability and translation beyond ribosomal substrates, potentially expanding NOP2’s influence on the cancer transcriptome [12, 45, 46].

Several limitations of this study should be acknowledged. First, our analyses relied exclusively on retrospective data from public databases, which preclude adjustment for treatment effects and other clinical confounders. In addition, because our survival analyses were primarily based on Kaplan–Meier comparisons and univariate HR estimates derived from public platforms, we were unable to determine whether NOP2 provides independent prognostic information beyond established clinicopathological or molecular biomarkers, or to evaluate its incremental predictive performance using metrics such as AUC or C-index. Second, the correlative nature of immune analyses cannot distinguish whether NOP2 expression actively shapes the tumor immune microenvironment or merely reflects underlying tumor biology. Third, the bulk RNA-seq data cannot resolve intratumoral heterogeneity or identify the specific cellular source of NOP2 expression. Finally, no *in vitro* or *in vivo* functional validation was performed in the present study; therefore, our data do not establish mechanistic links between NOP2 and immune modulation or tumor-promoting effects. Future studies incorporating gain- and loss-of-function experiments, immune co-culture systems, and where feasible, *in vivo* validation will be needed to clarify the biological and translational relevance of NOP2 in tumor immunity. Additionally, we acknowledge that several aspects of our analysis overlap with recently published studies examining NOP2 in pan-cancer contexts [19–21]. Nevertheless, our study provides independent validation of key findings while offering additional perspectives through integration of TCGA-GTEx datasets, analysis of RNA modification networks, and comprehensive immune checkpoint correlation analyses.

In conclusion, this pan-cancer analysis identifies NOP2 as a broadly dysregulated gene associated with prognosis and immune-related features across multiple human malignancies. These findings support further investigation of NOP2 as a candidate biomarker and potential therapeutic target, while underscoring the need for cancer-specific validation, multivariable prognostic modeling, and functional studies before clinical application can be inferred.

## Supplementary Material

Suppl 1 Differential expression levels of NOP2 across pathological stages in multiple tumor types. Differential expression levels of NOP2 at different pathological stages were analyzed in multiple tumor types in the pan-cancer cohort, including CESC, COAD, COADREAD, BRCA, ESCA, STES, and other tumor types.

Suppl 2 Top 100 NOP2-correlated genes identified in the GEPIA2 database.

## Data Availability

The authors declare that data supporting the findings of this study are available within the article and its supplementary information files. Any inquiries regarding supporting data availability of this study should be directed to the corresponding author.
