# Supplementary figures and images for: Pan-Cancer Analysis of NOP2 Reveals Its Prognostic Relevance and Association With the Tumor Immune Microenvironment
Source: World J Oncol. 2026 May 8;17(3):394–411. doi: 10.14740/wjon2739 (PMC13171267; doi:10.14740/wjon2739)

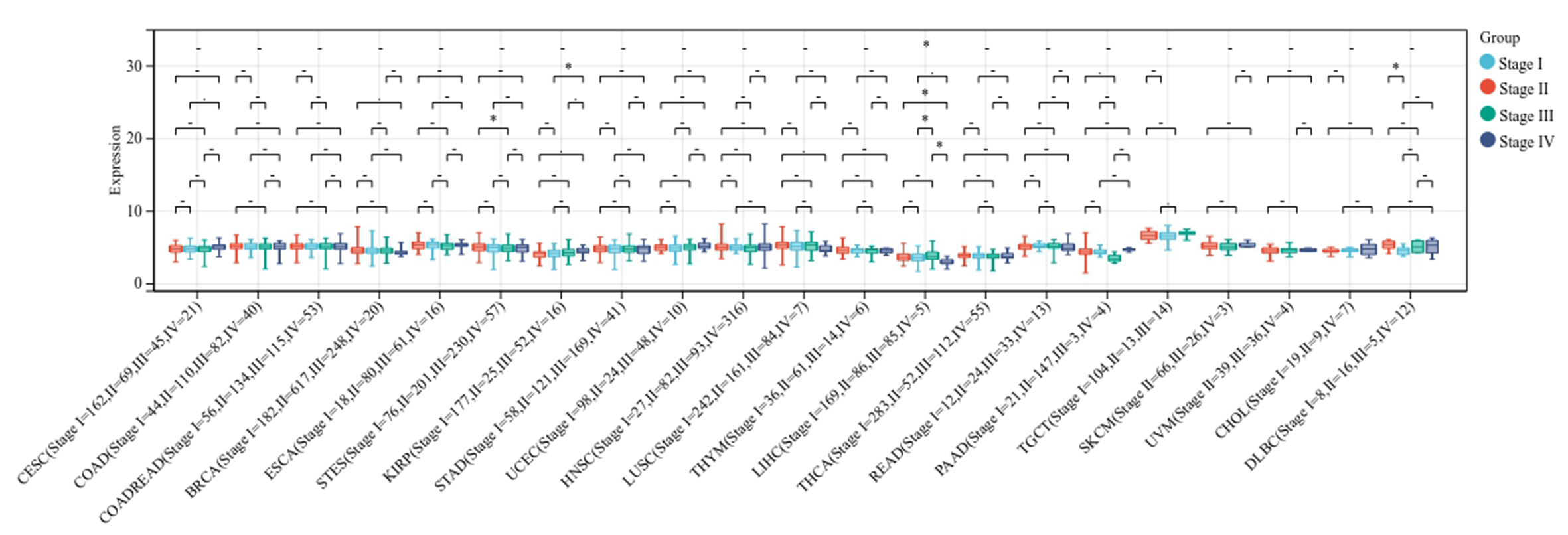

Supplement: Suppl 1 — Differential expression levels of NOP2 across pathological stages in multiple tumor types. Differential expression levels of NOP2 at different pathological stages were analyzed in multiple tumor types in the pan-cancer cohort, including CESC, COAD, COADREAD, BRCA, ESCA, STES, and other tumor types. [file wjon-17-03-394-s001.tif]
